# *Pseudomonas aeruginosa* Type III Secretory Toxin ExoU and Its Predicted Homologs

**DOI:** 10.3390/toxins8110307

**Published:** 2016-10-26

**Authors:** Teiji Sawa, Saeko Hamaoka, Mao Kinoshita, Atsushi Kainuma, Yoshifumi Naito, Koichi Akiyama, Hideya Kato

**Affiliations:** Department of Anesthesiology, School of Medicine, Kyoto Prefectural University of Medicine, Kyoto 602-8566, Japan; saekoh@koto.kpu-m.ac.jp (S.H.); mao5615@koto.kpu-m.ac.jp (M.K.); atsu-k@koto.kpu-m.ac.jp (A.K.); ynaitoh@koto.kpu-m.ac.jp (Y.N.); kanaslike@yahoo.com (K.A.); hide-16@koto.kpu-m.ac.jp (H.K.)

**Keywords:** ExoU, patatin, phospholipase A_2_, *Pseudomonas aeruginosa*, type III secretion system

## Abstract

*Pseudomonas aeruginosa* ExoU, a type III secretory toxin and major virulence factor with patatin-like phospholipase activity, is responsible for acute lung injury and sepsis in immunocompromised patients. Through use of a recently updated bacterial genome database, protein sequences predicted to be homologous to *Ps. aeruginosa* ExoU were identified in 17 other *Pseudomonas* species (*Ps. fluorescens*, *Ps. lundensis*, *Ps. weihenstephanensis*, *Ps. marginalis, Ps. rhodesiae, Ps. synxantha*, *Ps. libanensis*, *Ps. extremaustralis*, *Ps. veronii*, *Ps. simiae*, *Ps. trivialis*, *Ps. tolaasii*, *Ps. orientalis*, *Ps. taetrolens*, *Ps. syringae*, *Ps. viridiflava*, and *Ps. cannabina*) and 8 Gram-negative bacteria from three other genera (*Photorhabdus*, *Aeromonas*, and *Paludibacterium*). In the alignment of the predicted primary amino acid sequences used for the phylogenetic analyses, both highly conserved and nonconserved parts of the toxin were discovered among the various species. Further comparative studies of the predicted ExoU homologs should provide us with more detailed information about the unique characteristics of the *Ps. aeruginosa* ExoU toxin.

## 1. Introduction

Bacterial pneumonia that occurs frequently in patients under artificial respiration is defined as ventilator-associated pneumonia (VAP) [1]. *Pseudomonas aeruginosa* (hereafter called *Ps. aeruginosa*) is one of the most frequent causative bacterial agents of VAP [2]. Additionally, when *Ps. aeruginosa* is the causative agent of pneumonia, acute lung injury and secondary sepsis can easily occur, leading to high fatality rates [3,4,5,6,7]. In animal models of *Ps. aeruginosa* pneumonia, once cytotoxic *Ps. aeruginosa* strains are administered to the lungs of an experimental animal, the animal immediately develops an acute lung injury followed by bacteremia and secondary sepsis [8,9,10]. Over the past 20 years, investigations to identify virulence factors and their related mechanisms of action in *Ps. aeruginosa* have led to the discovery of the type III secretory toxin, ExoU, as a major virulence factor responsible for acute lung injury and sepsis [10,11,12,13]. In addition, clinical associations with *Ps. aeruginosa* ExoU genotype isolates have recently been reported in various other infections, such as chronic otitis media, keratitis, burns and bloodstream infections, and skin infections in diabetic patients [14,15,16,17,18,19].

Until recently, there was no obvious homolog of ExoU, with the exception of *Rickettsia prowazekii* PR534 [20], and *Ps. aeruginosa* ExoU was considered to be virtually the sole patatin-like cytotoxin secreted by the type III secretory mechanism. However, recent advances in DNA sequencing technologies have facilitated identification of several predicted ExoU homologs in various bacterial species, and these homologs have been reported in the bacterial genome database of the National Center for Biotechnology Information [21]. In this review, our brief summary of the molecular biology of the ExoU cytotoxin as a patatin-like phospholipase (PLP) is augmented by a comparative study of the predicted ExoU homologs from various bacterial species.

## 2. Molecular Biology of the ExoU Cytotoxin

**Figure 1:**
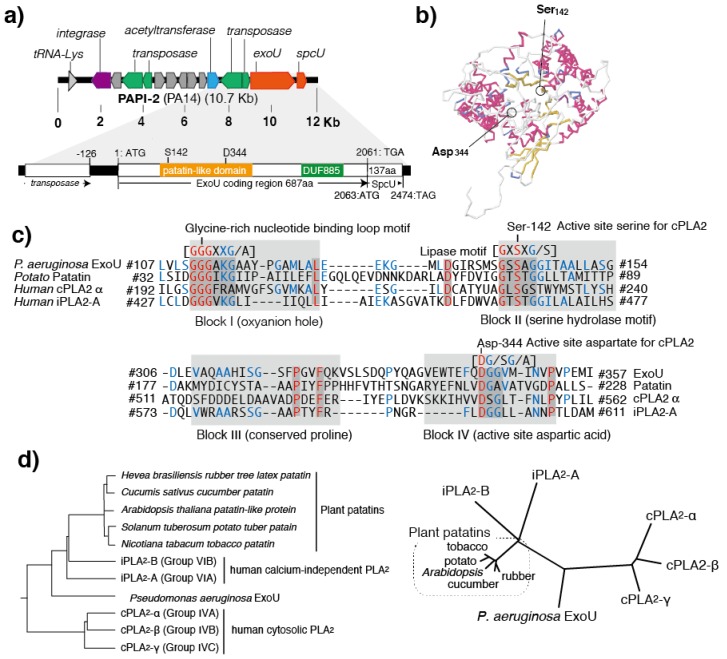
The structures of the ExoU gene and ExoU protein, and the phylogenetic trees based on patatin-like phospholipases (PLPs). (**a**) The position of the ExoU gene in the *Pseudomonas aeruginosa* pathogenicity island-2 (PAPI-2). PAPI-2 has 14 open read frames including *exoU* and *spcU*. The SpcU chaperone gene for ExoU secretion is located next to *exoU*. (**b**) Three-dimensional structure of ExoU (MMDB ID: 99839 PDB ID: 3TU3). The catalytic dyad consists of serine_142_ and aspartic acid_344_. (**c**) Amino acid sequence alignment of ExoU, patatins, and human cytosolic/calcium^2+^-independent phospholipase A_2_ (cPLA_2_/iPLA_2_). (**d**) ExoU, patatins, and human cPLA_2_/iPLA_2_ phylogenetic trees. Left, rooted tree with branch lengths; right, unrooted tree with branch lengths. The phylogenetic trees and the sequence alignments were made by ClustalW with the BLOSUM protein weight matrix. GenomeNet, Kyoto University Bioinformatics Center, Japan [35].

In 1997, ExoU was first identified in the cytotoxic *Ps. aeruginosa* strain that causes severe acute lung injury and sepsis in animal models of pneumonia [11]. In that study, the 2064-bp open reading frame of the ExoU gene (*exoU*) was cloned, and the predicted 687 amino acid protein encoded by it was characterized (Figure 1a) [11]. The TGA stop codon of *exoU* overlaps with the ATG start codon of *spcU,* the latter of which encodes the SpcU chaperone for ExoU secretion consisting of 137 amino acids [22]. ExoU was simultaneously characterized as one of the type III secreted toxins that *Ps. aeruginosa* injects directly into the cytosol of targeted eukaryotic cells through its type III secretory apparatus [11]. Later, clinical studies revealed that *Ps. aeruginosa* isolates possessing *exoU* cause bacteremia, sepsis, and high mortality [3]. Genetic analyses also have shown that the *exoU* gene is located in the *Ps. aeruginosa* pathogenicity island-2, and was found as an insertional gene cluster in the chromosomal genome of cytotoxic *Ps. aeruginosa* isolates (Figure 1a) [23].

Virulent *Ps. aeruginosa* isolates secreting ExoU show cytotoxicity in cultured epithelial cells and macrophages [9,10,11]. In various animal models, ExoU has been characterized as a major virulence factor for acute lung injury [10,11,12]. Among the various phenotypes of *Ps. aeruginosa* isolates, the ExoU-positive phenotype is a major risk factor for poor clinical outcomes [3,4]. A correlation between the antimicrobial characteristics of the bacterium and an *exoU*-positive genotype has also been reported in recent clinical studies [13]. After its discovery in 1997, the mechanism of acute cell death induced by the ExoU cytotoxin remained unknown for some time. However, in 2003, the PLP structure and the enzymatic activity of activated ExoU were elucidated [24]. Both in vitro and in vivo studies have shown that the phospholipase A_2_ and lysophospholipase activities of ExoU are activated by unknown eukaryotic cell factor(s) [25,26]. The PLP domain of ExoU contains a catalytic dyad consisting of serine (Ser_142_) and aspartic acid (Asp_344_) residues (Figure 1a,b) [24]. This catalytic dyad structure is similar to those of mammalian cytosolic phospholipase A_2_ (cPLA_2_) and calcium^2+^-independent phospholipase A_2_ (iPLA2), both of which belong to a family of PLP-domain proteins (Figure 1c) [24]. In ExoU mutant strains, which were genetically modified by introducing missense point-mutations into the catalytic dyad, there was a loss of PLP activity and cytotoxicity to eukaryotic cells [24]. Therefore, the PLP activity of ExoU was characterized as a cell death factor that acts to destroy cellular lipid membranes [24].

One feature of the type III secretion system is that the enzymatic actions of each toxin it contains are activated by eukaryotic cell factor(s) after the toxins are translocated into the cytoplasm of the targeted eukaryotic cell. For example, the ADP-ribosyl transferase activity of the *Ps. aeruginosa* type III secretory toxin ExoS is activated by a factor that is a member of the 14-3-3 protein family [27], while the adenylate cyclase activity of ExoY is activated by unknown factor(s) derived from eukaryotic cells [28]. Similarly, the PLA_2_ action of ExoU requires the presence of an activator contained in the cytosol extract of eukaryotic cells. In 2006, ExoU was reported to be activated by superoxide dismutase (SOD1) [29]. In the same year, other research reported that lysine_178_ and the carboxyl-terminal amino acids 679–683 of SOD1 are ubiquitinated during the activation process [30]. Subsequent studies revealed that SOD1 acts as the ubiquitin donor, and the ubiquitination of the carboxyl terminal domain of ExoU is the mechanism of PLP activation [31,32]. The carboxyl terminal of ExoU contains a conserved domain called DUF885, and mutant proteins lacking this region cannot be activated [31,32]. The three-dimensional structural analysis using crystallized cPLA2 revealed that serine and asparagine residues in the enzymatic center of PLP are located very close to each other and form a catalytic dyad in the three-dimensional structure [33]. However, structural analysis of the nonactive form of ExoU revealed that Ser_142_ and Asp_344_, which form the catalytic dyad for PLP, are separated further away from each other in the predicted three-dimensional structure of the nonactivated ExoU [34]. Therefore, it can be assumed that ubiquitination of the carboxyl-terminal brings Ser_142_ and Asp_344_ closer to each other to produce an active form of the dyad structure.

## 3. ExoU and PLP A2 Activity

Patatins, which account for about 40% of the soluble protein in potatoes (*Solanum tuberosum*) and are present in cucumbers (*Cucumis sativus*) and Para rubber trees (*Hevea brasiliensis*), are plant storage glycoproteins with acyl hydrolase activities [36,37]. Patatins are also known to be cross-reactive latex allergens that can cause latex allergies (natural rubber fruit syndrome, latex-fruit syndrome). Conversely, it has been found that many latex allergens contain Hev b7, a protein with a structure similar to patatin [38,39]. Patatin exhibits insecticidal activity against plant pest larvae; it is also considered to be an immune system protein of the plant kingdom, and has become known as an infection-specific pathogenesis-related protein in the plant pathology field [40].

Lipid-degrading enzymes generally possess the triad structure characterized by the presence of asparagine (or glutamic acid), serine, and histidine. However, unlike lipid-degrading enzymes, PLPs such as potato patatin B2, human cPLA_2_/iPLA_2_, and *Ps. aeruginosa* ExoU all generate their acyl hydrolase activities through the enzyme active site of the dyad formed by serine and asparagine [36]. There is a possibility that, during the evolutionary process, the gene encoding the patatin-like protein with insecticidal activity in plants was somehow incorporated into the genomes of pathogenic bacteria through a horizontal gene transfer mechanism. Indeed, *R. prowazekii* RP534—which was the only obviously recognized homolog of ExoU until recently [20]—has a PLP structure, and the recombinant protein of RP534 has PLA_2_ and lysophospholipase activities. Therefore, there is a possibility that *Rickettsia* RP534 is the link between plant and bacterial patatins [20].

## 4. Conserved Domains in PLPs

The PLP protein family, which includes ExoU, contains four conserved domains (blocks I–IV) (Figure 1c) [41,42]. Block I is a glycine-rich region containing an arginine or lysine residue and consisting of an oxyanion hole. Block II, which is located 10–20 amino acids away from block I, contains a GxSxG (or GxSxS) hydrolase motif sequence and a serine residue at the PLP enzymatic activity center. Block III contains several well-conserved proline residues in the group, and these are considered to be critical for maintaining the three-dimensional structure. In block III, the bacterial PLPs have an ASxxxP sequence, while the eukaryotic PLPs have an AAP sequence. Block IV contains an Asp-residue that is a pair component of a catalytic dyad with the Ser-residue of block I.

The patatin-like motif in ExoU is located at amino acid positions 110–354 in the amino-terminal side of the protein. First, the 110–125 position in block I of ExoU has an oxyanion hole (a glycine-rich nucleotide binding loop) comprising a GGGAxxG/A motif. The 139–145 position of ExoU block II has a hydrolase motif GxSxG (or GxSxS) with a catalytic center Ser_142_ residue. ExoU block III contains a patatin-specific proline at position 320 within the 309–325 amino acid region. Position 341–352 of ExoU block IV contains a D-G/S-G/A motif containing the other catalytic center residue, Asp_344_. This block displays high sequence homology with the blocks of other PLPs (Figure 1c). In the phylogenetic diagram that was created based on the primary amino acid sequence of the region containing blocks I and II of the patatin-like domain, ExoU is located just between human cPLA_2_/iPLA_2_ and a plant PLP group (Figure 1d).

## 5. Patatins and Phospholipases

**Figure 2:**
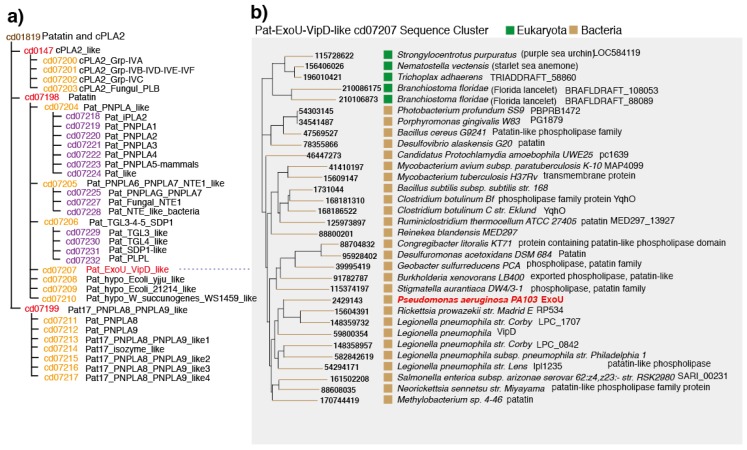
The conserved domain of patatins and cPLA_2_ according to the conserved domain database of the National Center for Biology Information [42]. (**a**) Patatin and cPLA_2_ domain (cd01819); (**b**) patatin and ExoU-VipD-like sequence clusters (cd07207).

The patatin and cPLA_2_ domain family (cd01819) consists of various patatin glycoproteins from plants and mammalian cPLA_2_ proteins (EC 3.1.1.4) (Figure 2a). The three major subdomains in these proteins are cPLA_2__like (cd0127), patatin (cd07198), and Pat17_PNPA8_PNPLA9_like (cd07199). The phospholipase A_2_ group of proteins is characterized by a lipid hydrolase catalytic dyad containing an active site serine and asparagine. cPLA_2_, which belongs to the domain family cd0147, is a group IV PLA_2_ that contains six intercellular enzymes (alpha, beta, gamma, delta, epsilon, and zeta). Vertebrate iPLA_2_ belongs to the domain family Pat17_PNPA8_PNPLA9_like (cd07199).

The Pat_ExoU_VipD_like domain family, which is one of seven patatin domain families, has a sequence cluster comprising ExoU of *Ps. aeruginosa*, VipD of *Legionella pneumophila,* several other *L. pneumophila* proteins, and proteins from *Mycobacterium*, *Clostridium botulinum*, *Burkholderia xenovorans,* and *Salmonella enterica* (Figure 2a). In addition, this family includes eukaryotic proteins from purple sea urchin, starlet sea anemone, *Trichoplax adhaerens*, and Florida *lancelet*. VipD was shown to have no phospholipase activity, even though it shares high sequence similarity with several functional regions of ExoU (e.g., oxyanion hole, active site serine, active site asparagine). Additionally, in the patatin-domain protein family, the closest homolog to *Ps. aeruginosa* ExoU is *R. prowazekii* PR534.

## 6. Predicted ExoU Homologs in *Pseudomonas* and Other Gram-Negative Bacteria

**Figure 3:**
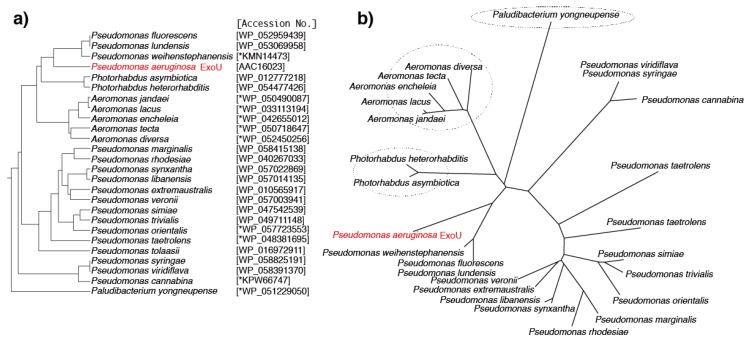
Phylogenetic trees of predicted ExoU homologs from the National Center for Biology Information GenBank database. (**a**) Rooted phylogenetic tree with branch lengths; (**b**) an unrooted phylogenetic tree with branch lengths. The phylogenetic trees were made by ClustalW with the BLOSUM protein weight matrix. GenomeNet, Kyoto University Bioinformatics Center, Japan [35]. * The ORF positions in GenBank were corrected in this study.

In our current search of the NCBI bacterial genome database we identified many predicted protein homologs of *Ps. aeruginosa* ExoU: 17 proteins from other *Pseudomonas* species and 8 proteins from other Gram-negative bacteria (Table 1, Figure 3a,b). *Photorhabdus* species are Gram-negative entomopathogenic bacteria of the family Enterobacteriaceae. Predicted ExoU homologs have been found in two *Photorhabdus* species. Among several members of this genus, *Photorhabdus asymbiotica* is a pathogen of both insects and humans. *Ph. heterorhabditis,* which was recently proposed to be a novel species of the genus *Photorhabdus*, was isolated from populations of the pathogenic insect nematode *Heterorhabditis zealandica* collected in South Africa [43]. It was reported that *Ph. asymbiotica* induces cellular death through the activation of caspases 8 and 9 and the executioner caspases 3 and 7 [44,45,46,47], and this suggests that the predicted ExoU homolog might be involved in the cell death mechanism.

*Aeromonas*, a Gram-negative, facultative anaerobic and rod-shaped bacterium, is ubiquitous in fresh and brackish water [48]. Most *Aeromonas* species are known to be associated with human diseases. Predicted ExoU homologs were found in five *Aeromonas* species. *Aeromonas jandaei* is a Gram-negative bacterium isolated from human feces in Oregon, USA [49]. *A. lacus* is one of three groups of *Aeromonas* strains isolated from lakes in Finland [50], and while *A. encheleia* was isolated from European eels in Valencia, Spain [51,52,53], *A. tecta* is a strain isolated from the stool of a child with diarrhea, from a healthy patient, and from environmental sources [54].

**Figure 4:**
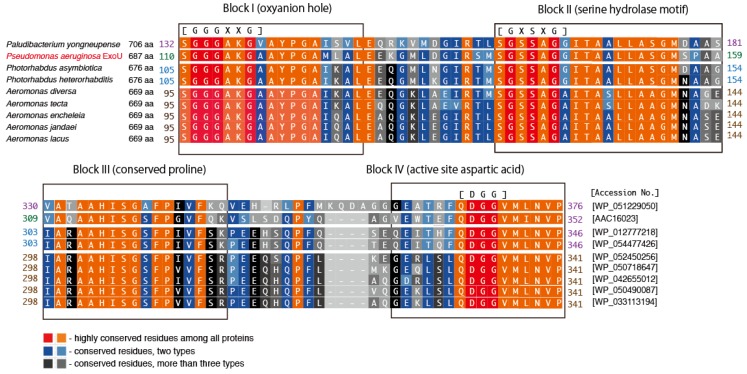
Conserved blocks in the PLP domain of predicted ExoU homologs from *Pseudomonas aeruginosa* and other bacterial species. The sequence alignment was made by ClustalW with the BLOSUM protein weight matrix (DNA Data Bank of Japan) [48].

*Paludibacterium yongneupense*, a bacterium that falls within the family *Neisseriaceae*, was isolated from a Korean wetland area [55]. In the primary sequence of the predicted *Pa. yongneupense* ExoU, blocks I–IV of this ExoU homolog are a good match with those of *Ps. aeruginosa* ExoU. Figure 4 shows the amino acid sequences of eight predicted ExoU homologs from various species aligned against the *Ps. aeruginosa* ExoU sequence. The predicted ExoU homologs from the three non-*Pseudomonas* species (*Paludibacterium*, *Photorhabdus*, and *Aeromonas*) exhibit high sequence conservation in blocks I–IV of the ExoU patatin domain, implying that they should probably exhibit phospholipase A_2_ activities once they are properly secreted and activated in the targeted eukaryotic cells, although their functionalities have not been tested yet.

**Figure 5:**
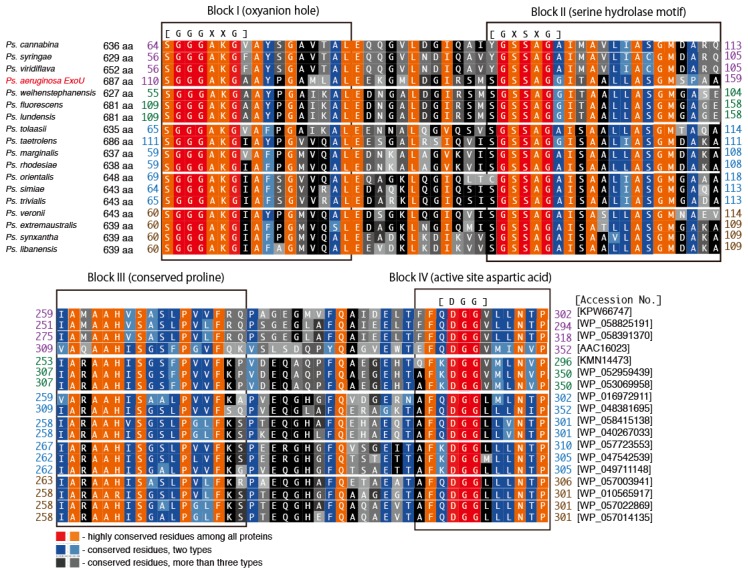
Conserved blocks in the PLP domain of predicted ExoU homologs from *Pseudomonas* species. The sequence alignment was made by ClustalW with the BLOSUM protein weight matrix (DNA Data Bank of Japan) [48].

Predicted *Ps. aeruginosa* ExoU homologs were found in the following 17 *Pseudomonas* species: *Ps. fluorescens, Ps. lundensis*, *Ps. weihenstephanensis*, *Ps. marginalis*, *Ps. rhodesiae*, *Ps. synxantha*, *Ps. libanensis*, *Ps. extremaustralis*, *Ps. veronii*, *Ps. simiae*, *Ps. trivialis*, *Ps. orientalis*, *Ps. tolaasii*, *Ps. taetrolens*, *Ps. syringae*, *Ps. Viridiflava*, and *Ps. cannabina*. The primary amino acid sequence alignment scores for these homologs against *Ps. aeruginosa* ExoU varied from 41% to 60%, as shown in Table 1. We previously investigated the polymorphism levels in four toxin genes, *exoS*, *exoT*, *exoU*, and *exoY*, of the *P aeruginosa* type III secretion system in several clinical isolates. We reported that ExoU, the largest of the toxin genes (2064 base pairs long encoding a 687 amino acid protein), is the most highly conserved of these toxins at the nucleic acid and amino acid sequence levels [56]. When compared with the PA103 reference strain, ExoU gene single nucleotide polymorphisms (SNPs) are scattered throughout the nucleotide sequence, but there are no changes in the 359 amino acid *N*-terminal sequence of the protein encoded by it. Furthermore, the five amino acid sequence alterations found in the *C*-terminal portion of the protein occur in only 16% of the strains that were sequenced. Therefore, the sequence variations in the ExoU homologs among *Ps. aeruginosa* ExoU and other Pseudomonad homologs are definitely much greater than those among clinical isolates of *Ps. aeruginosa*. However, as shown in Figure 5, the amino acid sequences of four key blocks in the predicted PLP domain of the 17 predicted ExoU homologs from *Pseudomonas* species are quite conserved, implying that these homologs should act as phospholipase A_2_ molecules once they are properly activated via the type III secretion mechanism.

**Figure 6:**
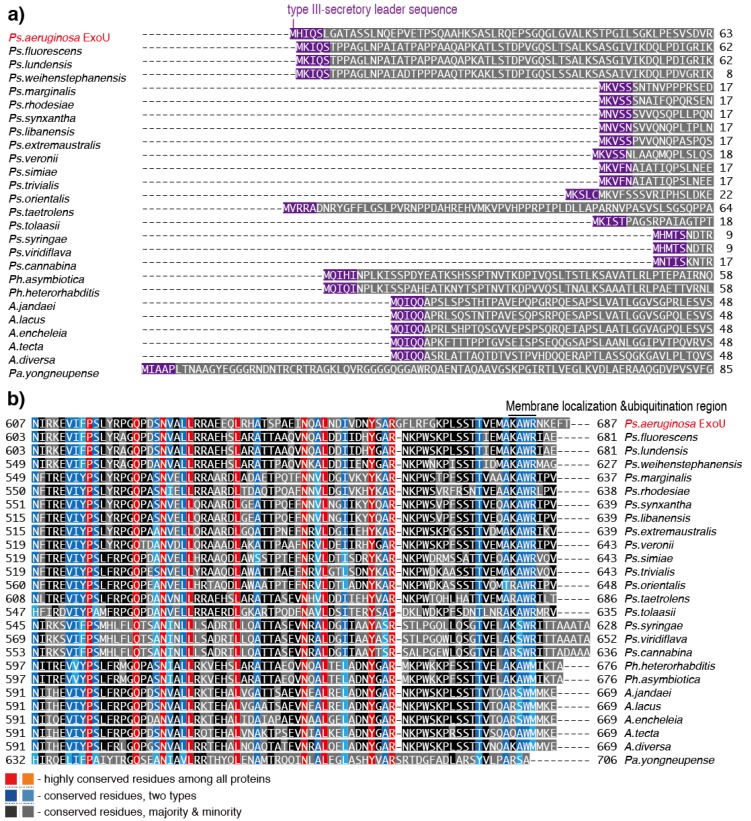
Sequence alignment of predicted ExoU homologs. (**a**) The amino-terminal alignment for predicted ExoU homologs; (**b**) the carboxyl-terminal alignment for predicted ExoU homologs. The sequence alignment was made by ClustalW with the BLOSUM protein weight matrix (DNA Data Bank of Japan) [48].

The predicted ExoU homologs from *Pseudomonas* genera, with the exception of *Ps. fluorescens, Ps. lundensis, Ps. weihenstephanensis, and Ps. taetrolens*, are 43–55 amino acids shorter at their amino-termini than that of *Ps. aeruginosa* ExoU (Figure 6a). The amino termini of the predicted homologs from *Ps. fluorescens*, *Ps. lundensis,* and *Ps. weihenstephanensis* each have a leader sequence (MKIQS) that is quite similar to the type III secretory leader sequence (MHIQS) of *Ps. aeruginosa* ExoU (Figure 6a). In contrast, the N-termini of the predicted homologs of other *Pseudomonas* species contain different leader sequence types such as MxVxx and MHxxS; however, we do not know whether they function as type III secretory leaders at this point in time.

Notably, amino acid sequence alignments show that the N-termini of the predicted ExoU homologs of *Photorhabdus* and *Aeromonas* contain ExoU-like leader sequences (MQIxI or MQIQQ), suggesting that these predicted proteins are probably secreted through the type III secretion mechanism (Figure 6a). In their C-termini, some variations are apparent in the primary sequences of the predicted ExoU homologs among all 22 bacterial species, although conserved regions exist among these homologs. Because the carboxyl terminal of ExoU is reported to be associated with enzymatic activation, further comparative analyses will help to resolve questions about the activation mechanism of this protein (Figure 6b). Such analyses are important because, until recently, no ExoU homologs had been tested for their potential in vitro enzymatic or cytotoxic activities against eukaryotic cells. Therefore, in the absence of information about such activities, we will need to wait for cytotoxicity testing of the ExoU homologs to be conducted to learn more about these proteins.

## 7. Predicted Homologs of the *Ps. aeruginosa* ExoU Chaperone, SpcU

**Figure 7:**
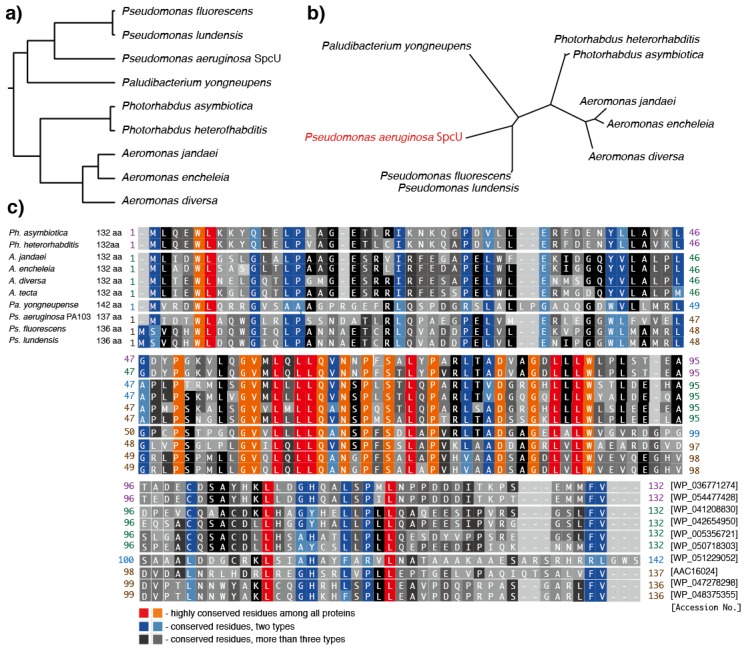
Sequence alignment of predicted SpcU homologs. (**a**) Rooted phylogenetic tree with branch lengths of the predicted SpcU homologs; (**b**) unrooted phylogenetic tree with branch lengths of the predicted SpcU homologs; (**c**) sequence alignment of predicted SpcU homologs. The phylogenetic trees and the sequence alignment were made by ClustalW with the BLOSUM protein weight matrix (DNA Data Bank of Japan) [48].

*Ps. aeruginosa* possesses the ExoU-specific chaperone protein, SpcU [22]. The gene for SpcU (*spcU*) is located just downstream of *exoU* (Figure 1a). Therefore, detecting the presence or absence of specific chaperones for each predicted ExoU homolog might help to determine whether the homologs are type III secreted or not. In the NCBI bacterial genome database, eight predicted homologs of SpcU were found in *Ps fluorescens*, *Ps. lundensis*, *Ph. heterorhabditis*, *Ph. asymbioitica*, *A. jandaei*, *A. encheleia*, *A. diversa*, and *Pa. yongneupense,* (Table 2, Figure 7). SpcU homologs have well-conserved primary sequences, especially in the PLP blocks of the central regions. The positions corresponding to ExoU Lys_178_, which is a known ubiquitination region, are conserved in all *Aeromonas* species and four *Pseudomonas* species, but not in the remaining six *Pseudomonas* and *Paludibacterium* species. The region corresponding to the ExoU carboxyl terminal residues 679–683, which has also been reported as the region ubiquitinated for enzyme activation, is quite well-conserved among all the predicted homologs.

## 8. Conclusions

ExoU, a major virulence factor in cytotoxic *Ps. aeruginosa*, has gradually revealed its unique characteristics since its discovery. There are still many unsolved questions about the ExoU toxin, such as its evolutionary origin, its association with its chaperone, the activation mechanism underlying its PLP activity, and the association between its PLP activity and the cell death mechanism. In this review, we analyzed the primary sequences of eight predicted ExoU homologs from Gram-negative bacterial species, and 17 predicted homologs from *Pseudomonas* species other than *Ps. aeruginosa*. Both conserved and nonconserved regions were identified in the multiple alignments of the predicted primary sequences of the putative homologs. At this moment in time, the knowledge gap concerning the enzymatic functionality and cytotoxicity of the predicted homologs of ExoU will remain in place until in vitro and/or in vivo analyses are conducted on these proteins. Nevertheless, in the near future, we can probably obtain more knowledge about the unique characteristics of ExoU from cloning the genes from the predicted ExoU homologs of *Ps. aeruginosa*, from gene complementation studies, and investigation of the characteristics of recombinant proteins that share sequence homology with ExoU.

## Figures and Tables

**Table 1:** Predicted ExoU homologs.

Species	NCBI GenBank Accession No.		DNA Sequence Length (bp)	Protein Sequence Length (aa)	Predicted Protein Size (kDa)	Alignment Score against ExoU (%)
	Protein	Genome				
*Pseudomonas aeruginosa* (ExoU)	AAC16023	U97065	2064	687	73.9	-
*Pseudomonas weihenstephanensis*	*KMN14473	JYLF01000003	2046	681	72.9	60
*Pseudomonas fluorescens*	WP_052959439	NZ_LCYS01000046	2046	681	72.7	60
*Pseudomonas lundensis*	WP_053069958	NZ_JYKY01000004	2046	681	72.7	59
*Pseudomonas marginalis*	WP_058415138	NZ_LKGY01000112	1914	637	67.9	43
*Pseudomonas rhodesiae*	WP_040267033	NZ_CCYI01000012	1917	638	68.7	42
*Pseudomonas synxantha*	WP_057022869	NZ_JYLJ01000004	1920	639	68.9	45
*Pseudomonas libanensis*	WP_057014135	NZ_JYLH01000017	1920	639	69.2	45
*Pseudomonas extremaustralis*	WP_010565917	NZ_AHIP01000021	1920	639	68.1	44
*Pseudomonas veronii*	WP_057003941	NZ_JYLL01000003	1932	643	69.1	45
*Pseudomonas simiae*	WP_047542539	NZ_JRMC01000004	1932	643	69.2	44
*Pseudomonas trivialis*	WP_049711148	NZ_CP011507	1932	643	69.0	44
*Pseudomonas orientalis*	*WP_057723553	NZ_JYLM01000004	1947	648	70.0	41
*Pseudomonas taetrolens*	*WP_048381695	NZ_JYLA01000004	2061	686	74.2	41
*Pseudomonas tolaasii*	WP_016972911	NZ_AJXG01000265	1911	635	68.4	42
*Pseudomonas syringae*	WP_058825191	NZ_LKEM01000062	1890	629	68.1	46
*Pseudomonas viridiflava*	WP_058391370	NZ_LKEJ01000112	1959	652	70.6	44
*Pseudomonas cannabina*	*KPW66747	LJPX01000541	1887	628	68.1	45
*Photorhabdus asymbiotica*	WP_012777218	NC_012962	2061	676	73.5	52
*Photorhabdus heterorhabditis*	WP_054477426	NZ_LJCS01000013	2061	676	73.7	51
*Aeromonas jandaei*	*WP_050490087	NZ_JFDL01000003	2010	669	71.1	48
*Aeromonas lacus*	*WP_033113194	NZ_JRGM01000022	2010	669	71.2	48
*Aeromonas encheleia*	*WP_042655012	NZ_CDDI01000033	2010	669	71.2	48
*Aeromonas tecta*	*WP_050718647	NZ_CDCA01000025	2010	669	71.5	46
*Aeromonas diversa*	*WP_052450256	NZ_CDCE01000029	2010	669	71.7	47
*Paludibacterium yongneupense*	*WP_051229050	NZ_AUGZ01000008	2121	706	74.3	45

Alignment scores (as based on ExoU amino acid sequence similarities according to the BLOSUM protein weight matrix) are from the ClustalW multiple sequence alignment tool (DNA Data Bank of Japan) [48]. bp: base pairs, aa: amino acids, * The open reading frame (ORF) positions in GenBank were corrected in this study.

**Table 2:** Predicted SpcU homologs.

Species	NCBI GenBank Accession No.		DNA Sequence Length (bp)	Protein Sequence Length (aa)	Predicted Protein Size (kDa)	Alignment Score against SpcU (%)
	Protein	Genome				
*Pseudomonas aeruginosa* (SpcU)	AAC16024	U97065	414	137	14.9	-
*Pseudomonas fluorescens*	WP_047278298	NZ_LCYS01000046	411	136	14.9	48.5294
*Pseudomonas lundensis*	WP_048375355	NZ_JYKY01000004	411	136	14.9	47.7941
*Photorhabdus asymbiotica*	WP_036771274	NZ_JONO01000032	399	132	14.8	31.8182
*Photorhabdus heterorhabditis*	WP_054477428	NZ_LJCS01000013	399	132	14.9	31.0606
*Aeromonas jandaei*	WP_041208830	NZ_JFDL01000003	399	132	14.4	28.7879
*Aeromonas encheleia*	WP_042654950	NZ_CDDI01000033	399	132	14.2	31.0606
*Aeromonas tecta*	WP_050718303	NZ_CDCA01000025	399	132	14.6	28.0303
*Aeromonas diversa*	WP_005356721	NZ_CDCE01000029	399	132	14.4	28.7879
*Paludibacterium yongneupense*	WP_050718303	NZ_AUGZ01000008	429	142	15.0	33.5766

Alignment scores (as based on SpcU amino acid sequence similarities according to the BLOSUM protein weight matrix) are from the ClustalW multiple sequence alignment tool with the BLOSUM protein weight matrix (DNA Data Bank of Japan) [48]. bp: base pairs, aa: amino acids.
